# Efficacy and Safety of Remdesivir in COVID-19 Positive Dialysis Patients

**DOI:** 10.3390/antibiotics11020156

**Published:** 2022-01-25

**Authors:** Batool Butt, Tajamul Hussain, Mu’taman Jarrar, Kashaf Khalid, Waleed Albaker, Asma Ambreen, Yasir Waheed

**Affiliations:** 1 Department of Medicine, Foundation University Islamabad, Islamabad 44000, Pakistan; batoolbutt7@gmail.com; 2 Research Chair for Biomedical Application of Nanomaterials, Biochemistry Department, College of Science, King Saud University, Riyadh 11451, Saudi Arabia; thussain@ksu.edu.sa; 3 Center of Excellence in Biotechnology Research, College of Science, King Saud University, Riyadh 11451, Saudi Arabia; 4 Vice Deanship for Quality and Development, College of Medicine, Imam Abdulrahman Bin Faisal University, Dammam 34212, Saudi Arabia; mkjarrar@iau.edu.sa; 5 Medical Education Department, King Fahd Hospital of the University, Al-Khobar 34445, Saudi Arabia; 6 Multidisciplinary Laboratory, Foundation University Islamabad, Islamabad 44000, Pakistan; kashaf.khalid1997@gmail.com; 7 Department of Internal Medicine, College of Medicine, Imam Abdulrahman Bin Faisal University, Dammam 34212, Saudi Arabia; wialbakr@iau.edu.sa; 8 Department of Medicine, Fauji Foundation Hospital, Rawalpindi 45000, Pakistan; asmayasir2005@hotmail.com

**Keywords:** COVID-19, hemodialysis, remdesivir, ESRD

## Abstract

(1) Background: Immune compromised hemodialysis patients are more likely to develop COVID-19 infections, which increase the risk of mortality. The benefits of Remdesivir, despite less literature support on its effectiveness in dialysis patients due to renal toxicity, can outweigh the risks if prescribed early. The aim of this study was to evaluate the efficacy of Remdesivir on the 30-day in-hospital clinical outcome of hemodialysis population with COVID-19 infection and safety endpoints of adverse events. (2) Study design: A prospective quasi-experimental study design was used in the study. (3) Methods: The sample population consisted of 83 dialysis patients with COVID-19 who were administered Remdesivir at a dose of 100 mg before hemodialysis, as per hospital protocol. After the treatment with Remdesivir, we assessed the outcomes across two endpoints, namely primary (surviving vs. dying) as well as clinical and biochemical changes (ferritin, liver function test, C-reactive protein, oxygen requirements, and lactate dehydrogenase levels) and secondary (adverse effects, such as diarrhea, rise in ALT). In Kaplan–Meier analysis, the survival probabilities were compared between patients who received Remdesivir within 48 h of diagnosis and those who received it after 48 h. Cox regression analysis was employed to determine the predictors of outcome. (4) Results: Of the 83 patients, 91.5% survived and 8.4% died. Remdesivir administration did not reduce the death rate overall. Hospital stays were shorter (*p* = 0.03) and a nasopharyngeal swab for COVID-19 was negative earlier (*p* = 0.001) in survivors who had received Remdesivir within 48 h of diagnosis compared to those who had received Remdesivir after 48 h. The only variables linked to the 30-day mortality were serum CRP (*p* = 0.028) and TLC (*p* = 0.013). No major adverse consequences were observed with Remdesivir. (5) Conclusions: Remdesivir has the potential to shorten the recovery time for dialysis patients if taken within 48 h of onset of symptoms, without any adverse effects.

## 1. Introduction

Coronavirus disease 2019 (COVID-19), declared as a global pandemic by the World Health Organization on 11 March 2020, is causing widespread havoc in healthcare sectors, resulting in about 318.6 million cases and 5.5 million deaths by 17 January 2022 [1]. COVID-19 affects 3.3% of the dialysis population, which is significantly higher than 0.2% of non-dialysis patients. In addition, the risk to those receiving hemodialysis in dialysis centers is two times greater than those enjoying home dialysis [2]. Evidence suggests that elderly people, especially those with a compromised immune system and multiple comorbid conditions, are more likely to be affected by COVID-19 infections [3]. Earlier studies showed that patients who undergo hemodialysis are immune compromised, have multiple conditions, such as heart disease, high blood pressure, diabetes, and lung disease, and are in a crowded health-care facility, with a higher risk of infection, resulting in adverse outcomes [3,4]. An alarmingly high level of mortality (20%), as compared to the general population has been identified in COVID-19 hemodialysis patients [5].

A variety of treatment regimens for COVID-19 infection have been tested by different research groups. During the first peak of COVID-19, there was only cogent evidence for corticosteroids in reducing the mortality rate [6,7]. The large randomized clinical trial executed in the UK validated the survival benefit of Dexamethasone in oxygen-dependent patients (23.3% vs. 26.2%; rate ratio 0.82; 95%CI, 0.72–0.94) [7]. In later years, several other drugs including Remdesivir, Tocilizumab or Baricitinib, and Favipiravir were approved by the FDA. However, the majority of drugs failed to demonstrate their worth in combating COVID-19 [8]. Recently, new oral drugs (Paxlovid and Molnupiravir) have been hailed by the FDA as the path forward in fighting COVID-19. Yet, not all patients have access to these drugs due to logistic reasons [9,10]. Several studies have failed to demonstrate the mortality benefit of Tocilizumab in COVID-19 patients [11]. However, the RECOVERY trial demonstrated a reduction in mortality and less need for mechanical ventilation among those treated with Tocilizumab [12].

Remdesivir, the first antiviral drug approved by the FDA in 2020 as an emergency treatment for moderate-to-severe COVID-19 infections, acts by inhibiting the RNA-dependent RNA polymerase (RdRp), which is associated with SARS-CoV-2 [13]. Initially, this drug was thought to be associated with less morbidity and mortality benefit. However, the two largest trials (Solidarity and ACTT-1) later challenged these claims [14,15]. Beigel et al. reported 20% clinical recovery with Remdesivir compared to the placebo group (RR 1.20; 95%CI, 1.12–1.27) [13]. The mechanistic insight of Remdesivir on dialysis patients will pave the way for researchers to cast around for effective COVID-19 drugs.

COVID-19 continues to be managed largely by supportive measures in hemodialysis patients due to the renal excretion of the majority of drugs used for treatment, as well as the lack of information on their efficacy and safety, which makes the outcome improvement difficult [16]. Paxlovid, Favipravir, and Remdesivir are renally excreted and not indicated in patients with a glomerular filtration rate (GFR) of less than 30 mL/min or in a dialysis population [8]. Although Molnupiravir is not excreted through the kidney [9], it is currently unavailable in Pakistan. In addition, Tocilizumab is a holy grail due to its cost and specific indications for COVID-19 [17,18].

While some studies do not support the effectiveness of Remdesivir among COVID-19 hemodialysis patients, it could be used to reduce the morbidity rate of COVID-19 in developing countries [19,20], where hospitals often lack the infrastructure and therapeutic options to treat COVID-19 pandemic patients. The immune response to vaccination in immune compromised dialysis populations is also less when compared to the general population. Therefore, the present study was undertaken to evaluate the efficacy of Remdesivir on the 30-day in-hospital clinical outcome of hemodialysis population with COVID-19 infection. Safety endpoints of adverse events were also assessed. This study is the first of its kind in Pakistan. With only a few published internationally [16,21], the study opens a door for researchers to discover effective treatment regimens, ultimately helping in curbing the spread of this fatal COVID-19 trajectory.

## 2. Materials and Methods

This non-randomized interventional study was conducted in compliance with the ethical standards at a tertiary care hospital from September 2020 to November 2021.

### 2.1. Study Cohort and Inclusion Criteria

**Figure 1:**
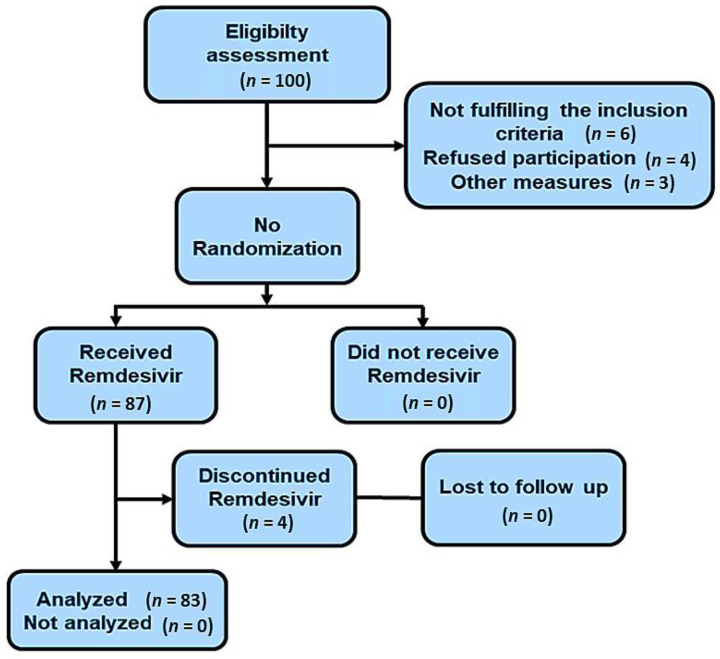
Flowchart for a single-arm, open label interventional study on Remdesivir for COVID-19 in hemodialysis population.

A total of 100 dialysis patients with COVID-19 infection were recruited to take part in the study. Six patients did not fulfill the inclusion criteria, 4 patients refused to participate, and 3 patients expired before the completion of Remdesivir therapy, leaving 87 patients in the study, as shown in Figure 1. The inclusion criteria comprised dialysis patients with COVID-19 infection who tested positive for SARS-CoV-2 within 10 days prior to the study. In addition, the inclusion criteria comprised one of the following criteria: Viral pneumonia on chest HRCT scan, patient’s oxygen saturation ≤ 94% on room air or requirement for oxygen or mechanical ventilation.

### 2.2. Exclusion Criteria

In this study, patients younger than 18 years of age, those with GFR > 30 mL/min, with abnormal liver function tests or alanine aminotransferase (ALT) levels exceeding five times upper limits of normality (ULN), as well as those not providing consent for participation were excluded.

Remdesivir was administered according to the institutional protocol. All of the patients received 100 mg for 5 days. The Remdesivir treatment could be extended to 10 days depending on the response. In addition, all of the patients received unfractionated Heparin 5000 units subcutaneously twice a day, injection of Dexamethasone 6 mg intravenously for 5 days with an extension up to 10 days depending on the clinical severity, followed by slow taper in a 2-week time frame and a broad spectrum antibiotic to cover the superadded bacterial infection.

### 2.3. Study Method

The study was conducted after obtaining the Ethics Committee’s approval and 83 patients were enrolled after receiving written informed consent from the patient or his/her first-degree relatives (in severely ill patients). Socio-demographic variables such as age, sex, duration, and frequency of dialysis; cause of end stage renal disease (ESRD); laboratory parameters (complete blood picture, serum creatinine, serum ferritin, serum ALT, C-reactive protein (CRP)); and lactate dehydrogenase (LDH) levels were included. A chest HRCT scan was advised for all of the patients and staging depended on the detection of parenchymal involvement. Hemodialysis was conducted every 48–72 h, depending on the clinical response and biochemical parameters. Nasopharyngeal swabs for SARS-CoV-2 by reverse transcription polymerase chain reaction were repeated on the 7th day after admission, and then repeated every 72 h until the test was negative. The discharge criteria included clinical improvement along with two negative nasopharyngeal swabs for SARS-CoV-2.

### 2.4. Statistical Analysis

Statistical analysis was performed using the Statistical Package for Social Sciences’ version 26.0. Qualitative variables were expressed as absolute numbers and percentages. Quantitative variables were expressed as the mean, standard deviation or as the median and interquartile range (IQR). The chi-square test for qualitative data, the independent samples t-test for continuous variables, and the Mann–Whitney U test for nonparametric data were used. A paired sample t-test was used to compare all of the parameters (continuous variables) before and after the Remdesivir treatment. A *p* < 0.05 was considered statistically significant. Kaplan–Meier and log-rank analyses were used for assessing the 30-day survival in hemodialysis population [22]. Moreover, the multivariate Cox proportional hazard (HR) regression model was used for analyzing the factors associated with mortality.

## 3. Results

Eighty-three dialysis patients with COVID-19 infection were recruited to participate in the study. Table 1 shows the characteristics, including the clinical and laboratory features of the study population. The mean age was 59.43 ± 14.28 years and the extremes were 22 to 70 years. The age group of 55–70 years represented 60% of the cases. Diabetes was the most common cause of ESRD, comprising about 34.1% of the cases. Fifty-one patients (60%) received Remdesivir within 2 days, of which 47 patients (92.1%) recovered while 4 (7.8%) died. Thirty-two patients (38.55%) received Remdesivir after 2 days, of which 90.6% (*n* = 29) survived and 12.5% (*n* = 4) died. Twenty-nine patients received five doses of Remdesivir, while in 4 patients Remdesivir was withheld due to the rise in ALT/aspartate aminotransferase (AST) values. In addition, in two patients, seven doses of Remdesivir were administered.

**Figure 2:**
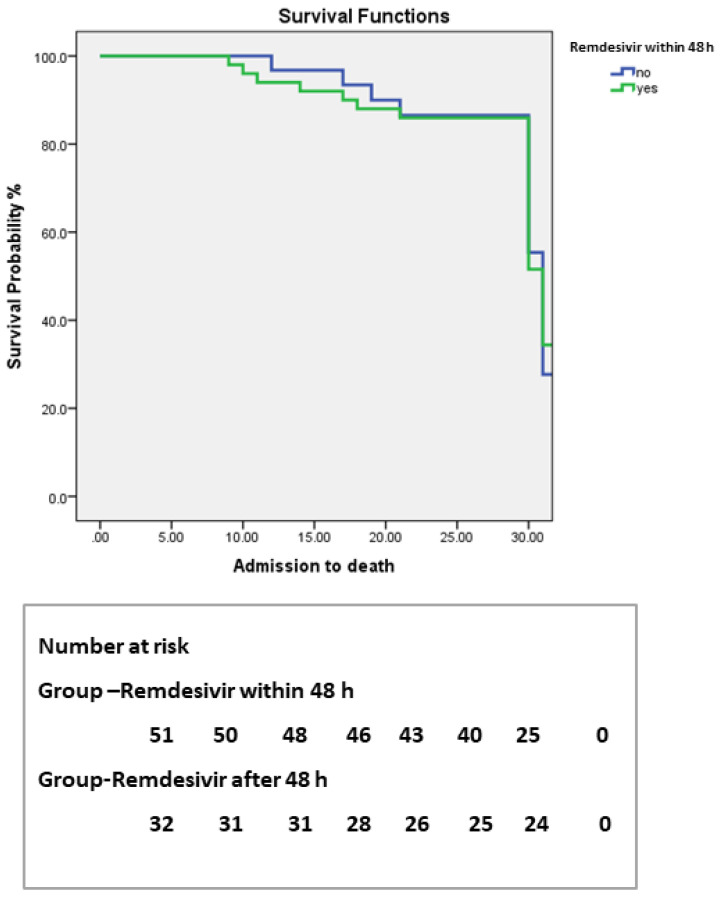
Kaplan–Meier analysis curve for 30-day survival probability from hospital admission of hemodialysis patients treated with Remdesivir within 48 h (green line) or after 48 h (blue line). The Kaplan–Meier analysis curve showed that the median time from hospital admission to death was 12 days for patients who received Remdesivir within 2 days (SE = 2.72, 95%CI = 9–12) compared to 9 days for patients who received it after 2 days (SE = 3.2, 95%CI = 7–9). The duration between the two groups was statistically insignificant (log rank χ^2^= 3.40, *p* < 0.065).

The primary endpoint of this study was to assess the efficacy of Remdesivir on the 30-day in-hospital clinical outcome of hemodialysis population with COVID-19 infection (survived vs. died). Of the 83 patients, 91.5% (*n* = 76) survived and 8.43% (*n* = 7) patients died. The mean duration of hospital stay was 9.59 ± 1.82 (6–14) days. The median time from hospital admission to death was 12 days for patients who received Remdesivir within 2 days (SE = 2.72, 95%CI = 9–12) compared to 9 days in patients who received Remdesivir after 2 days (SE = 3.2, 95%CI = 7–9). The duration between the two groups was statistically insignificant (Log rank χ^2^ = 3.40, *p* < 0.065). The Kaplan–Meier analysis curve showed the overall survival probability in relation to the use of Remdesivir within 48 h or receival after 48 h, as shown in Figure 2. Among survivors and in patients who received Remdesivir within 48 h, hospital stays were shorter with a median time of 9 days. In contrast, patients who received Remdesivir after 48 h had a median time of around 11 days (*p* = 0.03). Moreover, nasopharyngeal swabs for COVID-19 were negative in patients who received an early dose of Remdesivir compared to those who received it after 48 h (*p* = 0.02) (Table 2).

**Figure 3:**
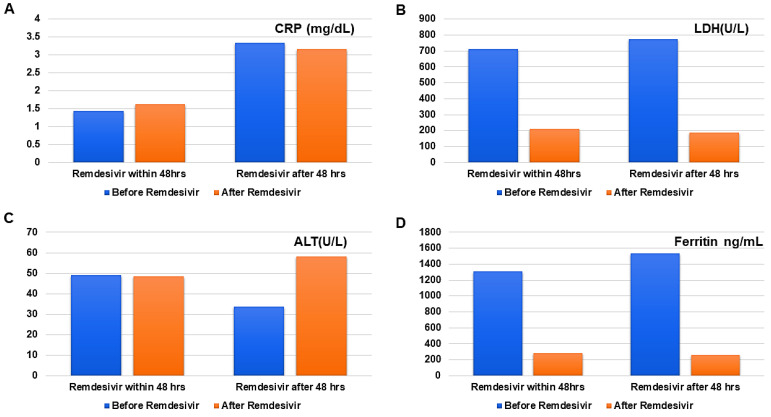
Comparison of biochemical parameters before (blue block) and after Remdesivir therapy (orange block). (**A**) CRP levels (mg/dL) before and after Remdesivir, (**B**) LDH levels decreased significantly after Remdesivir, (**C**) ALT levels were statistically insignificant, (**D**) Ferritin (ng/mL) showed a statistically significant decrease whether Remdesivir was administered within or after 48 h.

There was a significant decrease in ferritin and LDH levels, whether Remdesivir was administered within or after 48 h, and the results were statistically significant. However, no difference was observed in CRP levels (Table 3, Figure 3). Only 3 patients had increased ALT, while two patients experienced minor reactions (shivering and headache) after the drug was administered.

Finally, regression analysis was used to determine the factors affecting the mortality in COVID-19 positive dialysis patients. First, the factors were analyzed by linear regression. Then, only statistically significant factors were analyzed by multivariate Cox regression. Table 4 shows that only a raised CRP value and total leucocyte count were found to be associated with 30-day mortality.

## 4. Discussion

A review of the literature revealed that patients with comorbid conditions, such as diabetes, hypertension, and cardiovascular disease are more likely to develop a COVID-19 infection in a more severe form, requiring intensive-care unit (ICU) admission [23,24]. COVID-19 data are poor in dialysis population. In addition, the effectiveness of different treatment options for COVID-19 patients on dialysis is unknown. A study carried out in one hemodialysis center in Paris [25] and one in Wuhan [26] showed that patients undergoing hemodialysis are more likely to contract COVID-19 infection compared to others.

Remdesivir was the first FDA approved antiviral drug [27] for emergency use in moderate-to-severe COVID-19 infections. Initially considered a panacea for COVID-19, the drug was later found benign in only improving recovery times and shortening hospital stays, as evident by the two largest clinical trials conducted to date on Remdesivir, (Solidarity trial [14] and the ACTT-1 [15]). In contrast, a prospective double-blind trial conducted in China found no difference between Remdesivir and the placebo in early recovery [28].

There is a paucity of randomized controlled trials on the safety and efficacy of Remdesivir in patients with renal impairment having GFR < 30 mL/min due to devastating complications related to the prolonged half-life of the drug itself and its vehicle sulfobutylether-beta-cyclodextrin (SBECD) [29]. The presence of nephrotoxicity has been demonstrated in animal studies at doses 50–100 times greater than the dose used to treat the COVID-19 infection in humans with a 5–10-day course, which is quite low. Nonetheless, renal effects are rarely observed due to the very low doses accumulated with a 5–10-day course [30,31]. There is less mitochondrial toxicity and the products can be removed with dialysis. Even the randomized controlled trials conducted on Remdesivir in COVID-19 patients without renal impairment did not report any significant renal adverse events [32]. In this regard, patients should be given this drug since developing countries lack access to other drugs to counter this pandemic.

In this study, the mean age group was 59.43 ± 14.28 years, comprised mainly of females with no predilection for any age groups. This is in contrast to a previous study in which younger male patients with a mean age of 50.1 ± 12.2 years were primarily involved [33], signifying that all age groups can be affected due to the abated immunity and running dialysis in a closed environment. The female over-representation cannot be explained in the cohort, except for the fact that the dialysis center caters more to women due to their insurance. However, mortality is higher in men as apparent from the literature [34].

The most frequent symptoms reported in this cohort were dyspnea (91.5%) and fever (90.36%). This is similar to the general population [35], and is reported by the COVIDIAL study [36] as well as the China medical expert group. Both deceased and survivors had diabetes as the leading cause of kidney disease, which also adds a risk factor for increasing the likelihood of COVID-19 infection. The survivors had radiological evidence of COVID-19, including bilateral peripheral based ground-glass opacities in 19 patients (25%), crazy-paving appearance in 31 patients (40.7%), and bilateral consolidation in 14 patients (18.4%), compared to the deceased in which consolidation and ground-glass opacities predominate (28.57%). On the contrary, Ho Yuen Frank delineated consolidation as the ubiquitous finding (30 of 64; 47%) followed by ground-glass opacities (21 of 64; 33%) in COVID-19 patients [37].

All of the biochemical parameters including CRP, LDH, and ferritin are usually elevated in COVID-19 patients [13,38] and correlate with the severity of disease. A significant decrease in all of the parameters (except for the CRP level) occurs after Remdesivir therapy, whether it was initiated within or after 48 h. This difference can be attributed to the chronic inflammatory state in the dialysis population. Recent studies have brought to the fore a new concept of human oral microbiota and have tried to elucidate the relationship between oral dysbiosis (leading to increased inflammatory cytokines, including the CRP level which contributes to the higher SARS-CoV-2 viral load) and the increase in the severity of COVID-19 [39,40].

In this cohort, only CRP was found to be an independent predictor of mortality, analogous to the French and Spanish studies [41]. Moreover, the increased total leukocyte count provided a signal for poor outcomes (*p* = 0.01), similar to the general population [42,43]. However, no association was found between ALC and mortality as compared to the other studies [44].

The majority of the patients in this study were oxygen-dependent with 35 (42.1%) requiring an oxygen mask, 18 (21.6%) requiring a non-rebreathing mask, 18 (21.6%) CPAP, and 13 (15.3%) requiring mechanical ventilation. After Remdesivir therapy, 76 (91.5%) of the patients improved and maintained saturation in room air. This is in conformity to the study executed in a dialysis center in India and also another study conducted in non-dialysis patients with COVID-19 [45]. Mortality in patients who require mechanical ventilation is lower (14.28%) compared to the 75% mortality in ventilator patients, as delineated by Valeri A.M. et al. [46].

The current study showed that 51 (60.0%) of patients received Remdesivir within 2 days, which reduces the hospital stay by 3 to 4 days. This is in congruence to the largest multinational trial ACTT-1 conducted in a non-dialysis population and another study carried out in acute kidney injury (AKI) and dialysis-dependent patients [13,15]. Although the clinical and biochemical response of Remdesivir is more evident in patients with mild-to-moderate COVID-19 and those with lower oxygen requirements, its mortality benefit is not certain. Therefore, its use is not recommended for severe COVID-19 infection patients who require high oxygen support. In this cohort, a lower mortality rate (8.43%) was experienced compared to Chinese dialysis cohorts (14–31%) [47], Spanish dialysis units (23%) [48], and French dialysis units (24%) [28]. The reason was that the patients in this study had fewer comorbid conditions and the hospital, especially the ICU, was not overburdened. Four patients (57.1%) died in a Remdesivir group, in which the drug was initiated within 48 h and three patients (42.8%) after 48 h, with no significant difference.

No major untoward effects related to Remdesivir were observed in this study. This is in contradiction to the previous studies, which reported other side effects, such as diarrhea and rash [49,50]. Remdesivir elevated the ALT levels mildly in three patients (3.61%) after three doses, but remained static after five doses. In one patient (1.20%), where ALT levels increased significantly after three doses, Remdesivir had to be discontinued. These findings were similar to the study by Thakare et al. [32]. Additionally, in a study conducted by Pettit et al. at an academic medical center in Chicago, Illinois, a 10% discontinuing treatment was mentioned [51]. However, it is very difficult to discern whether the drug itself or COVID-19 infection engenders hepatotoxicity.

As this study covers a small sample of dialysis patients in a single center, there are no hard endpoints, such as a comparison with other treatment modalities or long-term effects of Remdesivir. Consequently, the conclusions that can be drawn from this study are limited. Therefore, we embolden further research in this field to confront all of these limitations and develop therapeutic innovations for improved clinical impact.

## 5. Conclusions

In conclusion, this study provided some evidence that the use of Remdesivir in CKD patients showed improved outcomes with timely initiation of treatment. A cataclysm and its consequences can occur due to COVID-19 infection in dialysis populations, resulting in high fatality rates. Furthermore, Remdesivir use in CKD patients should not obviate the necessity of administering Remdesivir to patients with COVID-19.

## Figures and Tables

**Table 1:** Baseline characteristics of the study population.

Parameters	Baseline *n* (%) (*n* = 83)	Discharge *n* (%) (*n* = 76)	Death *n* (%) (*n* = 7)	*p*-Value
Age (years)	59.43 ± 14.28	59.32 ± 62.50	60.57 ± 8.67	0.82
Sex (M/F)	31/50 (36.5/58.8)	27/47 (35.5/61.8)	4/3 (57.1%/42.8)	0.5
Cause of ESRD				
Diabetes	29/54 (34.1)	29 (38.1)	4 (57.1)	0.09
Hypertension	26/57 (30.6)	26 (34.2)	2 (28.57)	0.09
Others	26 (30.6)	21 (27.6)	1 (14.2)	0.08
Symptoms				
Fever	75/83 (90.3)	63/76 (82.89)	3/7 (42.8) (3)	0.03
Cough	66/83 (79.5)	68/76 (89)	7/7 (100)	1
Dyspnea–mild	76/83 (91.5)	70/76 (92.1)	6/7 (85.7)	0.42
Moderate	4/83 (4.8)	3/76 (3.9)	1/7 (14)	-
Severe	3/83 (3.6)		0	-
Symptoms’ duration before admission, days, median (IQR)	2 (1–5)	2.0 (1–5)	2 (1–4)	0.15
Hospitalization days, median (IQR)	9 (6–14)	9 (6–14)	11 (7–14)	0.06
Day of initiation of remdesivir, median (IQR)	2.0 (1–4)	1 (0–1)	1 (0–1)	0.14
Mean laboratory values				
NLR	13.22 ± 10.56	13.7 ± 11.41	11.2857 ± 5.96	0.93
ALT (U/L)	39.67 ± 46.71	37.96 ± 41.05	58.28 ± 91.17	0.32
Ferritin (ng/mL)	1395.57 ± 963.89	1449.47 ± 978.03	807.43 ± 547.52	0.06
LDH (U/L)	735.88 ± 522.96	735.93 ± 523.73	735.29 ± 555.831	0.27
CRP (mg/dL)	2.17 ± 2.57	1.99 ± 2.38	4.19 ± 4.07	0.44
ALC	897.08 ± 556.05	847.89 ± 542.06	1431.14 ± 435.211	0.05
CT scan of chest				
None	7 (8.2)	5 (6.5)	2 (28.57)	0.38
STAGE 1	21 (24.87)	19 (25)	2 (28.57)	
STAGE 2	32 (37.7)	31 (40.7)	1 (14.28)	
STAGE 3	16 (18.9)	14 (18.4)	2 (28.57)	
STAGE 4	7 (8.2)	7 (9.21)	0	
Oxygen requirement				
Oxygen mask	35 (42.1)	33 (43.4)	2 (28.5)	0.53
Non-rebreathing Mask	18 (21.6)	16 (21.0)	2 (28.5)	
CPAP	18 (21.6)	16 (21.0)	2 (28.5)	
Mechanical ventilation	12 (14.4)	11 (14.4)	1 (14.2)	

ESRD: End-stage renal disease; IQR: Interquartile range; NLR: Neutrophil to lymphocyte ratio; ALT: Alanine transaminase; LDH: Lactate dehydrogenase; CRP: C-reactive protein; ALC: Absolute lymphocyte count; CPAP: Continuous positive airway pressure.

**Table 2:** Comparison of parameters between patients who received Remdesivir before and after 48 h of admission.

Parameters	Initiation before 48 h (n–51)	Initiation after 48 h (n–32)	*p*-Value
Hospitalization days, median (IQR)	9 (7–11)	11 (9–12)	0.03
Days to swab negative, median (IQR)	7 (6–9)	9 (8–11)	0.02
Need of mechanical ventilation, median (IQR)	9 (7–11)	14 (11–14)	0.01
Death *n* (%)	4 (7.8)	4 (12.5)	0.8

**Table 3:** Comparison of biochemical parameters before and after Remdesivir therapy.

Parameters	Before Remdesivir	After Remdesivir	*p*-Value
Remdesivir initiated within 48 h			
ALT (U/L)	49.25 ± 59.84	48.411 ± 48.36	0.00
CRP (mg/dL)	1.43 ± 1.51	1.63± 3.45	0.90
Ferritin (ng/mL)	1310.33 ± 1067.05	281.91 ± 180.54	0.00
LDH (U/L)	711.31 ± 329.91	211.01 ± 76.49	0.00
Remdesivir initiated after 48 h			
ALT (U/L)	33.66 ± 35.53	58.25 ± 74.38	0.00
CRP (mg/dL)	3.34 ± 3.39	3.17 ± 3.46	0.89
LDH (U/L)	775.03 ± 735.44	187.37 ± 63.62	0.00
Ferritin (ng/mL)	1531.41 ± 768.20	256.87 ± 175.92	0.00

ALT: Alanine transaminase; CRP: C-reactive protein; LDH: Lactate dehydrogenase.

**Table 4:** Multivariate Cox regression analysis of factors affecting the mortality of hemodialysis patients with COVID-19.

Variable	HR	95%CI	*p*-Value
Age	1.00	0.041–25.0	0.99
Remdesivir within 48 h	1.25	0.196–8.00	0.81
Number of days requiring oxygen	0.42	0.018–10.13	0.59
Comorbid conditions (diabetes, hypertension)	1.00	0.046–21.91	0.99
Number of days on ventilation	0.90	0.033–24.49	0.95
ALC	1.00	1.000–1.00	0.10
TLC	1.17	1.035–1.33	0.01
CRP	1.00	1.000–1.00	0.03

HR: Hazard ratio; CI: Confidence interval; NIV: Non-invasive ventilation; ALC: Absolute lymphocyte count; TLC: Total leucocyte count; CRP: C-reactive protein.

## Data Availability

The data presented in this study are available on request from the corresponding author.
